# From toxicity to conformity: adaptive user behavior to social norms in Telegram communities

**DOI:** 10.1038/s41598-026-49756-w

**Published:** 2026-04-24

**Authors:** Lorenzo Alvisi, Victoria Popa, Guglielmo Cola, Serena Tardelli, Maurizio Tesconi

**Affiliations:** 1 Institute of Informatics and Telematics, National Research Council, Pisa, Italy; 2 IMT School for Advanced Studies Lucca, Lucca, Italy; 3 Department of Computer Science, University of Pisa, Pisa, Italy

**Keywords:** Mathematics and computing, Psychology, Psychology

## Abstract

Toxic and antisocial user behavior on social media platforms has received considerable scholarly attention due to its detrimental effects on society. This study takes a holistic perspective on the phenomenon of online toxicity by investigating the impact of local community norms on toxic expression. By using six large-scale datasets, comprising over 500 million Telegram messages collected between 2015 and 2024, we analyze toxic user behavior across multiple chats and languages. We introduce a methodological framework that uses a conformity index to characterize conformist, anti-conformist, and independent behavioral tendencies. Our findings show that most users tend to conform to local normative environments, aligning their toxicity with the toxicity levels of the chats in which they participate. This pattern is consistent across datasets and languages, showing a strong association between community norms and user behavior online. Furthermore, we observe that higher levels of user participation in chats are associated with a stronger tendency toward conformity with the surrounding social contexts. Collectively, these findings contribute to a deeper understanding of toxic online behavior and highlight the importance of contextualized approaches to content moderation.

## Introduction

Human behavior, to be comprehensively understood, must be examined in relation to the social contexts in which it occurs. Individuals do not act in isolation; instead, their opinions and actions are shaped by the surrounding environment, interpersonal relationships, and social norms. Social norms signal what is considered appropriate within a given context by conveying information about what others typically do (descriptive norms) and what behaviors are socially approved or sanctioned (injunctive norms), thereby guiding individual conduct^1^. Consequently, the context in which people interact exerts a substantial influence on their behavior. This principle, referred to as “situational power,” emphasizes the critical role of external factors and the surrounding social environment in shaping individuals’ thoughts, emotions, and actions^2,3^. Since social interaction is mirrored in digital spaces, research has focused on how offline behavioral dynamics translate online, where social media platforms strongly influence perceived norms^4,5^. As a result, studies show that social norms play a powerful yet double-edged role in shaping behavior and interaction within online communities^6,7^. While they can promote positive outcomes such as social cohesion and collective activism^8^, they can also contribute to polarization, exclusion, and the spread of toxic^5,9–11^ and conspiratorial behaviors^12–14^.

Within this broader context, the present study focuses on online toxicity. As a form of human behavior, online toxicity cannot be fully understood in isolation from its contextual setting; rather, it offers a valuable lens through which to gain insight into the underlying social norms and identity dynamics that characterize online communities. Toxicity in digital spaces is a prominent research topic, as detrimental behaviors such as harassment, hate speech, and incivility are pervasive across social media platforms, and increasingly affect the tone and quality of public conversations, degrading the health of online communities and the overall user experience^15^. Despite substantial academic and applied interest in the topic, the state of the art still faces significant challenges in defining online toxicity with precision, which in turn hinders its effective detection and mitigation through robust moderation strategies^16,17^. These issues stem from the absence of a universally agreed conceptual framework, leading to the broad grouping of diverse antisocial behaviors, such as incivility^18^, hate speech^15^, cyberbullying^19,20^, and harassment^15,21^ under the general umbrella term of toxicity.

In the present study, we conceptualize online toxicity as rude, disrespectful, or otherwise discouraging language in online discussions, including expressions that may lead participants to disengage from the conversation. This framing captures a broad category of antisocial communication that can encompass or overlap with the narrower forms of abuse mentioned above, without being limited to any single one of them. To operationalize this construct, we use the “toxicity” attribute of the Perspective API. This API is based on a supervised machine-learning model developed by Google’s Jigsaw and trained on large corpora of human-annotated online comments^22^. It supports multiple languages and provides several content attributes, including severe toxicity, identity attack, insult, profanity, and threat. In this study, we focus exclusively on the primary toxicity attribute in order to obtain a consistent operational measure of perceived toxicity across contexts, aligned with our broad conceptual definition. This attribute assigns each comment a continuous score between 0 and 1, estimating the likelihood that the comment will be perceived as toxic. Previous research has widely used the Perspective API toxicity score to examine online toxicity and antisocial behavior on social media platforms^23–26^.

Classical approaches in this area have focused mainly on the detection and mitigation of toxic behavior in online environments, with a strong emphasis on the analysis of text-level content^23,26–28^. While valuable, this approach can overlook the social dynamics within online communities that contribute to the persistence of toxicity at a broader scale. Sheth et al.^17^ highlight the limits of current approaches to identifying and addressing online toxicity, noting that these methods often focus narrowly on isolated posts or content, disregarding the broader systemic and contextual factors in which such toxicity occurs. In fact, research has also extended the focus to community-level dynamics^29,30^, shedding light on how community norms^28,31^, architecture of the platforms^32^, and content moderation policies^33,34^ can foster toxicity diffusion. Other studies have explored toxicity from a longitudinal perspective, examining how individual user behavior evolves over time and across communities, with the aim of characterizing the behavioral patterns associated with toxic engagement^24,35^. In general, the role of toxicity as a social norm and the normalization process of online toxic behavior have been explored most extensively within specific contexts such as online gaming communities, where toxic behaviors are more easily accepted and often embedded in group culture. Studies show that players may view toxic behavior as normalized^36^ and that exposure to it increases the likelihood of imitation and contagion^11^. Among the studies that explored user behavior and the influence of context, Rajadesingan et al.^37^ analyzed users on politically oriented subreddits on the Reddit platform. They showed that users quickly adapt to the specific community toxicity norms and do not carry over their behavior from one community to another. This finding is further supported by a previous study by Chandrasekharan et al.^6^ which showed that after Reddit banned hate subreddits, members of those places did not engage in similar hate speech in other subreddits they joined later.

Prior research has examined toxicity from multiple perspectives, yet cross-community analyses remain limited. Existing studies have primarily focused on platforms such as Reddit and X (formerly Twitter), while research on Telegram has mostly addressed toxicity as a peripheral issue within broader investigations of conspiracy communities and extremist discourse^38^. However, Telegram’s structural characteristics make it particularly suitable for examining toxic dynamics from a cross-community perspective. The platform is organized around group chats that function as relatively well-bounded communities, where distinct norms and interaction styles may emerge. Users often participate in multiple groups, allowing the same individuals to be observed across different community contexts. Moreover, Telegram is characterized by limited search and recommendation mechanisms, meaning that users must more actively decide which communities to join. As a result, observed behavior is less shaped by platform-driven curation, enabling more direct within-user and cross-community comparisons. This makes Telegram especially useful for examining whether toxicity reflects stable individual tendencies or shifts in response to distinct local norms, a comparison that is more difficult on algorithmically curated platforms such as X, where exposure to content and interactions is strongly mediated by recommendation systems. Together, these features make the platform especially well suited for investigating toxic dynamics across different normative environments.

In summary, despite extensive research on online toxicity, important gaps remain in the literature. In particular, much of the research focuses on isolated cases of highly toxic user behavior and on the importance of content moderation, with limited attention to how toxicity is influenced by context and local community norms. Studies addressing the social dimension of toxicity often examine specific contexts, such as gaming, without considering how individuals behave differently across communities and settings. As a result, cross-community perspectives remain underexplored. Building on these gaps, in this study we adopt a multidisciplinary and context-sensitive approach using Telegram data to examine how users’ toxic behavior aligns with or deviates from the normative pressure of different communities. In particular, we address two research questions. *RQ1* investigates whether users’ toxicity varies systematically with the aggregate toxicity of the chats in which they participate. *RQ2* examines whether, at the individual level, users tend to conform, anti-conform, or remain independent with respect to chat toxicity, and whether conformity is the predominant pattern. These three behavioral patterns (conformist, anti-conformist, and independent) are grounded in prior literature on social influence and conformity^39^. The research questions reflect our theoretical expectation that user behavior is context-dependent and shaped by local descriptive norms rather than simply reflecting stable individual dispositions. We further assess whether these patterns are consistent across multilingual settings.

To investigate these questions, we use toxicity levels as a proxy for behavioral patterns within chats and operationalize chat-level descriptive norms as the aggregate toxicity of each chat. This allows us to assess whether individual users’ toxicity varies across chats consistently with differences in their behavioral environments. Our analysis focuses on observed behavioral norms, as perceived and injunctive norms cannot be inferred from behavioral trace data alone. Specifically, we analyze users’ behavior across multiple chats in which they actively participate, accounting for both individual variation in toxicity and the overall toxicity of each chat, using six multilingual datasets comprising approximately 500 million messages. In addition, this work contributes a new large-scale Telegram dataset collected throughout 2024, containing over 200 million messages and forming a subset of the overall corpus used in the analysis. We examine four linguistic contexts to assess whether the association between user toxicity and chat-level toxicity generalizes across languages. Although baseline toxicity and linguistic expression may vary across contexts, a consistent pattern of normative alignment would suggest a broader underlying social process.

We show that, at a global level, user behavior is strongly correlated with the context in which individuals participate, with toxicity levels tending to align with the prevailing toxicity of each chat. This pattern is consistently observed across all analyzed datasets, suggesting a strong relationship between local community norms and user behavior in multilingual settings. In addition, we analyze individual user behavior in relation to the surrounding context and find that conformist users represent the majority, indicating that alignment with local norms is the dominant tendency in the studied communities.

## Results

### Preliminaries

#### Datasets

Our analysis draws on six Telegram datasets that cover various contexts and languages. This diversity enables the examination of whether users’ conformist tendencies and toxic behavior are consistent across different settings. Specifically, we use multiple datasets: a multilingual Telegram dataset^40^ that we subdivide into four language-specific subsets (i.e., English1, Russian, Italian1, Portuguese); a dataset of Italian Telegram messages leveraged in^41^ (i.e., Italian2); and an English dataset collected for this study (i.e., English2). These datasets show minor differences in data collection methodologies, as described in Section Methods. A detailed summary of dataset sizes is provided in Table 2. The datasets comprise only public Telegram channels and groups (excluding private one-to-one conversations), with the analysis focusing on groups (hereafter, chats), since we are interested in studying the relationship between users and their conversational environment.

**User selection** We restrict the dataset to user-chat pairs (*u*, *c*) such that user *u* sent at least 100 messages in chat *c*; we define this condition as being *active* in a chat. We further require that *u* is active in at least one additional chat, ensuring that each selected user is active in at least two distinct chats and thus participates in multiple interaction contexts. This criterion allows us to focus on active participants and to capture behavioral variation across different chats. The distribution of the number of chats per user is reported in Supplementary Figure S1, with summary statistics in Supplementary Table S1 and Table S2. Further details on the processed datasets are provided in Section Methods.

#### Toxicity metrics

To analyze expressions of toxicity and users’ behavioral shifts, we rely on a set of metrics. We employ the Perspective API^22^, a state-of-the-art validated tool for detecting toxic language. The API assigns each Telegram message a toxicity score between 0 and 1, with messages classified as toxic if the score exceeds a predefined threshold, as suggested by Perspective API documentation. Additional details are provided in Methods.

**Chat toxicity** We define the toxicity of a chat as the percentage of toxic messages within a given chat.

**User toxicity** For each user, we compute toxicity separately in each chat in which they are active, as the percentage of their messages in that chat that are classified as toxic. The same user may therefore have different toxicity values across chats, since this quantity is not computed cumulatively across all of their messages. A formal definition of the metrics used can be found in Methods.

**Fig. 1:**
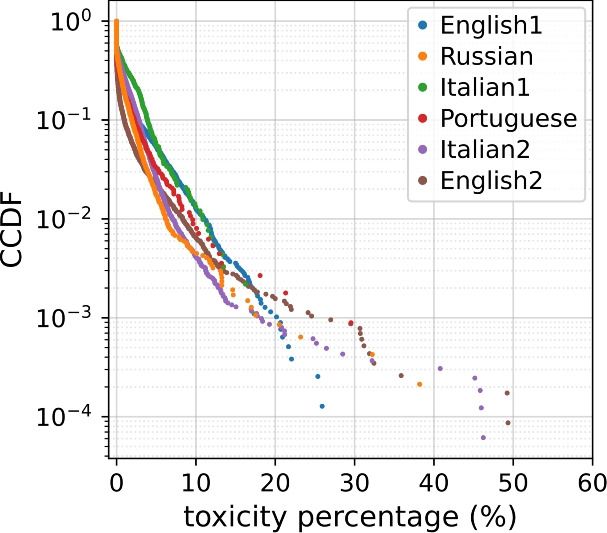
Complementary cumulative distribution function (CCDF) of chat toxicity across datasets. All datasets, despite linguistic and contextual differences, display similar tail behavior indicating the presence of both low-toxicity and highly toxic communities, providing variability for cross-environment behavioral analysis.

**Chat toxicity distribution** In Figure 1 we report the complementary cumulative distribution function (CCDF) of chat toxicity. All datasets display a clear tail behavior, either approximately exponential or heavier-than-exponential, consistent with the presence of long- or short-tailed regimes. This observation confirms that each dataset includes different levels of chat toxicity, an essential condition for examining variation in user behavior across different environments.

### Global patterns of adaptive user behavior

**Fig. 2:**
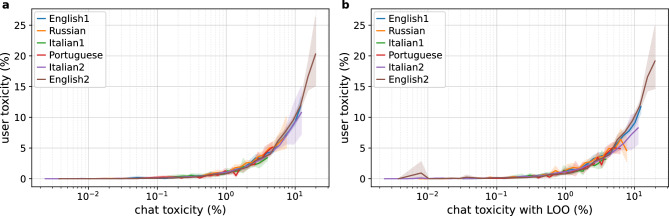
Correlation between user toxicity and chat toxicity. (**a**) The plot shows that, across all datasets, user toxicity increases with the toxicity level of the chat they participate in. The same pattern holds in (**b**) when removing the user’s own contributions (Leave-One-Out), confirming that the effect reflects genuine environmental influence. Shaded areas represent 95% confidence intervals. Correlation values are reported in Table 1.

Our first research question (RQ1) examines whether user toxicity varies with community-level toxicity across chats. We expect that context shapes user behavior, such that users tend to express higher toxicity in more toxic chat environments. To test this relationship, we compute the correlation between chat toxicity and user toxicity (Figure 2a). Since chat toxicity distributions display distinct tail behavior, we apply logarithmic binning to the data. This approach reduces noise and local fluctuations, enabling clearer visualization of global trends that are otherwise difficult to observe in the raw distribution. The effectiveness of logarithmic binning is supported in the literature^23,27,42–44^, and (log-)binned correlations have also been used in previous studies^23,45,46^.

As shown in Figure 2a, all datasets display a common increasing trend. We observe strong correlations between user toxicity and chat toxicity across all datasets and languages considered, indicating that users tend to be more toxic in chats characterized by higher overall toxicity levels. To further validate and strengthen this result, we implement a robustness check based on a leave-one-out framework. For this analysis, we consider the toxicity of the chat, for a given user, as the percentage of toxic messages within that chat, excluding the user’s own contributions. As such, the estimated chat toxicity captures only the surrounding environment and avoids user bias. As shown in Figure 2b, the consistency of the results across definitions further supports the conclusion that the observed association is not an artifact of the chosen metric. We further assess the robustness of these results with respect to variations in the number of bins and threshold values. The observed correlations remain stable across binning configurations (Table 4). Additional details are provided in the Methods section.

**Fig. 3:**
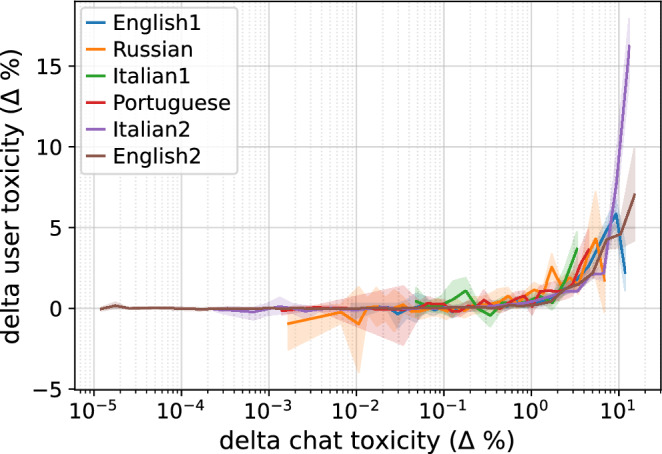
Variation in user toxicity as a function of the variation in chat toxicity. For every user active in at least two chats, the plot shows how variation in community toxicity corresponds to variation in the user’s own toxicity. All datasets display a clear positive trend: users tend to exhibit higher toxicity in more toxic environments. This dynamic evidence shows that user behavior is shaped by contextual norms rather than static personal tendencies.

The previous analyses do not take into account whether the same user engages in environments with different levels of toxicity. To address this, we test whether users’ toxicity varies with the surrounding environment, rather than simply reflecting self-selection^47^, whereby users choose to participate only in chats with preferred toxicity levels. Therefore, for each user, we consider the set of chats in which they are active and, for every pair of such chats, we define two quantities: the variation in chat toxicity, computed as the difference between the more toxic chat and the less toxic one (delta chat toxicity), and the corresponding variation in the user’s toxicity, defined as the difference between the user’s toxicity in the more toxic chat and in the less toxic one (delta user toxicity). By construction, the former is always non-negative, while the latter can take either positive or negative values. A more formal definition of this procedure is provided in the Methods section. This allows us to assess whether variation in user toxicity is associated with differences in the surrounding conversational environment. To reduce the noise, we applied the same binning methodology as in the previous analysis. The results, presented in Figure 3, show a consistent trend and strong correlations across all datasets, supported by statistically significant p-values. We additionally verify whether the quadratic growth in the number of chat pairs per user could cause the analysis to be dominated by a small fraction of users participating in many chats. We find that this is not the case: users participating in many chats have a limited relative influence on the pairwise analysis. The distribution of user participation and its contribution to the total number of chat pairs are described in Figure S1 and Table S3 of the Supplementary Information. This analysis further implies that users’ use of toxic language is strongly correlated with environmental conditions, showing that users tend to exhibit behavior aligned with those conditions across different contexts.

Finally, we test the robustness of these findings using a permutation-based null model. Specifically, we test the null hypothesis that the observed correlations arise from chance under independence by randomly permuting the chat toxicity values and repeating the analysis 1, 000 times. This procedure breaks the correspondence between user toxicity (or its variation) and chat toxicity (or their variation) while preserving the marginal distributions. Randomized analyses yield correlations centered around zero (as shown in Table 1), indicating that the empirical results depend on the original pairing between users and their conversational environments rather than on marginal distributions alone.

### Individual patterns of user norm conformity

**Fig. 4:**
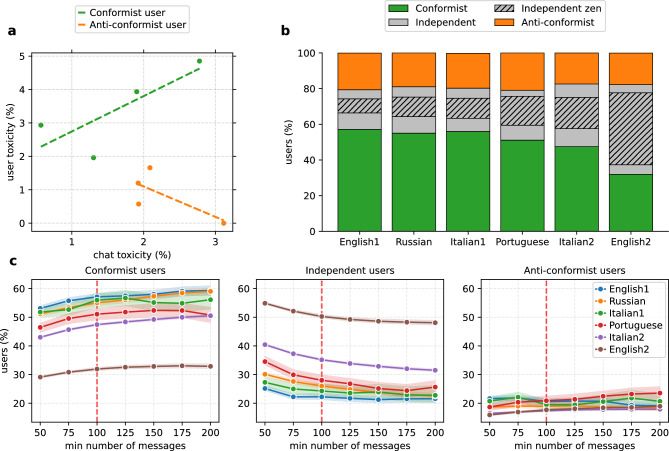
Individual patterns of conformity to community norms. (**a**) The plot depicts two examples of fitted user trends, showing how individual user-specific slopes capture different behavioral patterns across diverse chat environments. (**b**) shows the distribution of these behavioral types across datasets, where the majority of users appear to be conformists, with independents and anti-conformists representing smaller shares. This pattern remains consistent when tested across different slope thresholds (Figure 5). (**c**) shows how these proportions change when increasing the minimum number of messages required for inclusion. In particular, higher user activity and engagement are associated with more conformist behavioral patterns. Solid lines represent the observed values for each dataset, while shaded areas indicate the variability estimated via non-parametric bootstrap resampling (mean ± 1 standard deviation). The vertical red dashed line denotes the configuration used for (b).

So far, we have established that user toxicity is strongly associated with the toxicity of the surrounding environment across chats and within users. However, these analyses capture aggregate patterns and, consistent with the ecological fallacy^48^, cannot be used to infer individual behavioral tendencies. Therefore, even though we observe consistent global patterns between user and community toxicity, we cannot assume that individual users necessarily modify their behavior in response to contextual norms. To address this, our second research question (RQ2) investigates whether users tend to conform to, anti-conform with, or behave independently of the toxicity of the surrounding communities, and to what extent conformity represents the predominant pattern. To this end, we introduce an analytical framework that captures how users vary their toxicity in relation to the environmental level of toxicity within chats. For each user, we consider all chats in which they have posted, computing both the chat toxicity and the degree of toxic language they exhibit within that context. We fit a regression line through these points, extracting a slope and an intercept for each user. We conceptualize these slopes as a *conformity index*, capturing three types of user responses to contextual toxicity: *conformist* (positive slopes, indicating that user toxicity tends to align with the surrounding environment), *anti-conformist* (negative slopes, indicating that user toxicity tends to vary in the opposite direction), and *independent* (slopes near zero, indicating stability across different contexts and adherence to a consistent behavioral pattern). Another interesting finding emerges within the independent category. Notably, we identify a group of users who exhibit no toxicity in any context and consistently refrain from using toxic language. We refer to them as *zen users* to emphasize their consistent self-control. In summary, this methodology allows for the identification and classification of different behaviors emerging from observable data. The example in Figure 4a shows how the resulting slopes may vary between users, revealing heterogeneous and context-dependent online behavioral patterns.

In formalizing this methodological framework, we anchor it to existing theoretical work. In particular, our approach resonates with classical models of social psychology, and specifically with Willis’s tripartite model of social response^39^. This model considers conformity, independence, and anti-conformity as distinct dimensions of individual reactions to social influence. Although more recent and comprehensive psychological models also account for the interplay between public and private spheres in explaining individual conformity pressure^49^, the limitations of our dataset constrain our analysis to observable public behaviors within Telegram chats. The alignment between our empirical framework and Willis’s model provides a strong theoretical foundation to understand the behavioral patterns observed in our data. This correspondence supports our interpretation that user behavior is molded by contextual factors, with local community normative rules exerting a significant influence in shaping user conduct.

Given the heterogeneous behaviors observed among users, we further examine their predominant tendencies and distribution. Figure 4b shows the distribution of behavioral categories across datasets in a cross-sectional analysis across chats: in all cases, most users exhibit conformist behavior, aligning their toxicity with the surrounding environment. A smaller proportion of users can be classified as anti-conformist, whereas the share of independent users varies more substantially across datasets, reaching particularly high values in English2, a pattern that may partly reflect the dataset’s collection methodology and its thematic focus on cryptocurrency-related communities, whose interactions are often informational or promotional^50–52^ and thus tend to be less confrontational and less frequently classified as toxic^41^. Further discussion is provided in the Supplementary Information. Additional descriptive statistics and details for the behavioral categories across datasets are provided in Table S9 in the Supplementary Information.

To assess the robustness of the conformity index, we performed a leave-one-chat-out sensitivity analysis in which the conformity regression was refitted after removing one chat at a time for each user. The resulting behavioral classifications show high stability across datasets, particularly for conformist and anti-conformist users, while independent users exhibit lower stability, given that their classification corresponds to slopes close to zero; therefore, results for this group should be interpreted cautiously. Further details are reported in Table S8 in the Supplementary Information.

Another important aspect we aim to investigate concerns how users’ behavior varies with respect to the number of messages they sent in chats. As outlined in *Preliminaries*, our analysis considers users who posted at least one hundred messages across a minimum of two different chats each. Therefore, we study how the results in Figure 4b change as the minimum threshold of messages sent by users in chats varies. Figure 4c shows how the distribution of users across the three categories: conformists, anti-conformists, and independents varies as the minimum chat activity threshold increases from 50 to 200 messages. As thresholds rise, the share of independent users decreases, anti-conformists remain stable, and conformists become more prevalent. This indicates that higher levels of user activity are associated with a stronger alignment to community-level toxicity patterns.

**Table 1:** Correlations and baselines across datasets. Correlations measure the relationship between user and chat toxicity: (a) standard chat–user correlation (Figure 2a), (b) leave-one-out correlation excluding the user’s own messages (Figure 2b), and ($\Delta$) dynamic correlation between variations in user and chat toxicity (Figure 3). Baselines are computed by randomizing user–chat associations. Double asterisks (**) denote statistical significance ($p < 0.01$).

	**Correlations**			**Baselines**	
**Dataset**	Chat–User (Figure 2a)	LOO (Figure 2b)	Dynamic (Figure 3)	Random (Figure 2a)	Random (Figure 3)
English1	0.998**	0.996**	0.866**	$0.014 \pm 0.109$	$-0.053 \pm 0.373$
Russian	0.992**	0.952**	0.831**	$-0.057 \pm 0.284$	$-0.015 \pm 0.290$
Italian1	0.988**	0.980**	0.820**	$-0.019 \pm 0.218$	$0.001 \pm 0.306$
Portuguese	0.983**	0.974**	0.950**	$-0.008 \pm 0.173$	$0.006 \pm 0.277$
Italian2	0.999**	0.994**	0.928**	$-0.136 \pm 0.386$	$-0.040 \pm 0.409$
English2	0.999**	0.998**	0.993**	$0.000 \pm 0.230$	$0.001 \pm 0.280$

## Discussion

In this study, we show that users generally tend to align their behavior with the local context of the communities in which they participate, conforming to community-specific norms regarding toxic behavior. These findings support the view that online toxic dynamics are not solely attributable to stable individual traits, but are associated with the communicative environments in which interactions occur. This overall pattern emerges consistently across the Telegram communities examined, at both the global and individual levels and across all datasets and multilingual contexts considered in this work. At the global level, the strong correlation between user toxicity and chat toxicity underscores the importance of the broader communicative climate in shaping user behavior. At the individual level, the predominance of conformist behavior across datasets suggests that community norms may exert a substantial influence on online conduct, although anti-conformist and independent tendencies remain present. Notably, the positive association between higher activity levels and conformist tendencies further suggests that sustained participation in chat may strengthen alignment with local norms.

Overall, these findings show that toxic dynamics emerge as a context-dependent social phenomenon distributed across and shaped by community norms, conformity pressures, and group dynamics. Toxic behavior can therefore serve as an indicator of descriptive norms prevalent in certain communities, signaling what is commonly practiced and implicitly tolerated. However, toxicity alone does not allow us to determine whether such behaviors have also been internalized as injunctive norms and perceived as socially approved or expected. In some contexts, such as certain gaming communities, hostile interactions may nonetheless be perceived as normalized, potentially making toxic behavior appear acceptable or routine within the local interaction climate.

Our results underscore the multifaceted nature of online toxicity, which emerges from the combined influence of contextual and relational factors. This perspective aligns closely with insights from social psychology, particularly theories of normative influence and individual behavioral adaptation to conformity mechanisms. In particular, we observe a clear cross-community pattern in which user behavior is closely associated with the toxicity of the local context. We also consider the self-selection hypothesis, a mechanism widely discussed in the social and behavioral sciences whereby individuals tend to participate in environments that match their interests, preferences, or behavioral tendencies^47^. In the context of online chats, this mechanism would imply that users with a higher propensity for toxic behavior preferentially participate in communities where such behavior is already prevalent, while less toxic users gravitate toward less toxic environments^6,53^. Under a pure self-selection mechanism, users with different baseline tendencies would simply sort into communities with similar behavioral norms, resulting in relatively stable behavior across environments. However, our observations suggest that this explanation alone is insufficient. Many users participate in multiple chats with markedly different toxicity levels, with their toxicity tending to correspond to the local interaction climate. This suggests that the observed pattern reflects not only individual dispositions but also normative pressures operating within the community, consistent with prior research^37^. In fact, a substantial body of research has examined the dynamics of individual conformity to group norms and expectations^1,54–57^. As demonstrated in Asch’s conformity experiments^54^, collective pressure has the power to lead individuals to align themselves with the majority opinion, even though there is no real internal adherence. In fact, the desire to avoid disapproval or social exclusion appears to prevail more over personal judgment, highlighting the powerful role of normative influence^55^ in shaping individual behavior. This occurs even in situations where it is recognized that these norms are arbitrary^2^ or do not align with the actual beliefs of individuals^54,58^. This theory may provide a plausible explanation for why users generally tend to adjust their behavior to the specific communities they join.

Online communities likewise demonstrate the significant shaping force of social norms. According to the Identity Model of Deindividuation Effects (SIDE)^59^, anonymity, understood as reduced personal identifiability, enhances the salience of group social identities and individual conformity to group normative behavior. Consequently, individuals are more susceptible to group influence when their personal identity cues are limited^60^. In this regard, Telegram affords a relatively high degree of pseudonymity, limited identity verification, and is widely recognized as a privacy-oriented platform. These structural features may create contextual conditions in which group-level norms become more salient and more influential. Building on this framework, in groups where antagonistic or aggressive norms are already embedded, reduced identifiability may facilitate conformity to prevailing group expectations. In our context, this helps explain why toxic behavior often co-varies with community-level toxicity. In fact, users exposed to such environments may feel socially pressed to conform to locally dominant normative expectations that structure interaction within the group. Importantly, anonymity, conceptualized as reduced identifiability, does not by itself constitute a causal driver of toxicity, but it may influence the extent to which existing prosocial or antisocial group norms shape behavior. This interpretation reflects theoretical accounts emphasising multifactorial origins of online disinhibition^61^ and is supported by empirical evidence showing that toxic behavior emerges when anonymity co-occurs with additional situational factors such as invisibility and reduced social cues^62^.

Moreover, our findings suggest that online toxicity is not driven only by a small group of highly toxic users^30,63^. Instead, it is distributed across communities and shaped by collective and contextual dynamics that encourage conformity to local norms. In other words, when placed in certain environments, many users can display toxic behavior as a response to situational and normative pressures, regardless of their personal dispositions. This perspective challenges intervention strategies that rely primarily on identifying and removing toxic users or content, and instead highlights the need to address the broader community-level conditions that enable toxicity to persist^64^. Future research could further explore how individuals exhibiting high levels of toxic behavior, potentially associated with antisocial traits^65^, may relate to community-level normative climates. Moderation approaches should also consider the normative environment and contextual features of the communities in which users interact, as these factors play a meaningful role in shaping behavior. Therefore, since users tend to align their behavior with the norms of the communities in which they participate, moderation decisions should primarily be based on the conduct of the users within a specific community rather than on their past behavior elsewhere. Moreover, distinguishing between descriptive and injunctive norms is essential for understanding how toxic discourse takes shape within online communities. Even in chats where toxic behaviors are common as descriptive norms, strengthening injunctive norms that emphasize prosocial conduct through visible cues, clear rule reminders, or the amplification of constructive contributions can help reduce conformity to harmful patterns and promote healthier environments^66^. In fact, prior research has shown that combining injunctive and descriptive social norms results in the greatest improvement in the reporting behavior of users when combating fake news on social media^67^. In addition, future research might also test targeted social interventions, either with human participants or using LLM-based agents, such as introducing prosocial actors into highly toxic communities or simulated environments to assess whether proactive norm-seeding helps improve the local interaction climate.

We acknowledge that our study presents some limitations. Although our analyses are based on a large volume of user data, they are restricted to a single platform, namely Telegram. In particular, the structural features of Telegram, such as large group sizes, weak moderation, and limited accountability, may facilitate the persistence of toxic behavior by enabling harmful norms to become embedded within communities. As such, we should be cautious in generalizing our findings to other online environments, where platform design and moderation policies may shape user behavior differently. Future work should extend this analysis to other platforms to assess the extent to which these findings hold across platforms. Furthermore, classifier-based toxicity scores present important limitations, especially in multilingual settings. In the case of Perspective API, prior research has highlighted concerns related to contextual sensitivity, linguistic bias, and uneven performance across languages. Given the multilingual nature of our datasets, these factors may affect both the reliability of toxicity estimates and the cross-lingual comparability of the scores, since cultural and linguistic nuances may not be fully captured. Moreover, we note that Perspective API is no longer supported as of 2026, which may further limit its applicability in future research. In addition, the selected datasets may not be representative of Telegram communities in general. Finally, since the study is observational and correlational, it is not possible to determine to what extent contextual factors, such as chat topics, directly influence user behavior. Future research should explore how specific community dynamics influence toxic norms and incorporate temporal information, enabling a more detailed investigation of how user behavior evolves over time in response to changes in community norms.

## Methods

### Toxicity detection

The definition of online toxicity is a well-recognized challenge, as it is often used as an umbrella term that encompasses various forms of antisocial communication^23,68,69^. For this reason, researchers often rely on an operational definition, such as the one adopted by Perspective API^22^, which defines toxicity as “a rude, disrespectful, or unreasonable comment that is likely to make you leave a discussion”^22–24,70^.

However, the use of Perspective API to measure toxicity requires some methodological considerations. Since Perspective API is a general-purpose classifier, its toxicity scores may be sensitive to linguistic variation, and absolute score values may therefore not be fully comparable across heterogeneous contexts, as discussed in^71^. To account for this limitation, we perform all analyses separately within six language-specific datasets, rather than pooling raw scores across corpora. Moreover, following previous work^23^, we convert the continuous toxicity score into a binary toxic/non-toxic label using the threshold of 0.7 recommended in the Perspective API documentation. This reduces the impact of residual context-dependent variation in the continuous score, since small differences in score values may reflect differences in language use rather than substantial differences in perceived toxicity.

### Datasets and data collection

Our study relies on six Telegram datasets originating from three main data sources, each differing in collection methodology and linguistic scope, as described below.

- *The Pushshift Telegram Dataset*^40^: This large-scale multilingual Telegram dataset includes 96 million chats and 317 million messages collected between 2015 and 2019 using a snowball strategy. Since it includes messages from multiple languages and topics, we extracted four subsets for cross-linguistic comparison, which we refer to as English1, Italian1, Russian, and Portuguese.
- *The Italian Telegram Ecosystem Dataset*^41^: This dataset contains more than 186 million messages posted in Italian Telegram chats between January 1 and December 31, 2023. It was collected with an iterative snowball approach that began from a set of seed channels and expanded by following forwarded messages. We refer to it as Italian2.
- *Original English Telegram dataset collected for this study*: Our third dataset is an English-language Telegram dataset collected specifically for this study. Starting from the list of English Telegram channels available on tgstat.com, we retrieved all associated public group messages posted in 2024. This dataset differs slightly from the previous ones, as we did not employ an iterative snowball crawling approach. It contains more than 238 million messages, as detailed in Table 2. We refer to it as English2.

Overall, these datasets contain more than 522 million messages. For consistency, we refer to all language-specific subsets as separate datasets, as shown in Table 2. The inclusion of two English and two Italian datasets reflects their independent origins and data collection procedures. By combining datasets from prior literature with newly collected data, we are able to assess the robustness of our findings within the same linguistic context, across distinct samples. This is particularly important given the stringent requirement of at least 100 messages per user–chat pair, which substantially limits the number of eligible users and chats.

**Table 2:** Overview. Statistics of the datasets.

Dataset	Time span	Chats	Users	Messages
English1^40^	2015–2019	24,503	431,214	29,447,990
Russian^40^	2015–2019	11,346	342,619	48,569,460
Italian1^40^	2015–2019	6,536	103,892	8,928,252
Portuguese^40^	2015–2019	6,592	96,467	9,732,694
Italian2^41^	2023	15,871	1,307,169	186,809,126
English2	2024	12,074	9,691,170	238,923,774
**Total**	**-**	**-**	**-**	**522,411,287**

### Data preprocessing

To ensure the reliability of our analyses, we applied a multi-stage data processing pipeline designed to guarantee comparability across datasets and languages. First, we restricted the corpus to user-generated textual messages only, excluding system notifications, bot messages, automatic service messages, and any form of non-textual media (e.g., images, videos, stickers, GIFs, voice notes). Since Perspective API cannot label non-linguistic content or very short tokens, we additionally removed empty messages, messages containing only URLs, emojis, or non-recognized characters, as well as messages that the API failed to classify. Summary statistics of the preprocessing outcomes, reporting the number of messages retained after Perspective API labeling for each dataset, are reported in Table S7 of the Supplementary Information.

Given the centrality of user-level behavioral comparisons across multiple communities, we imposed strict filtering criteria on user activity. Specifically, we included only those users who produced at least one hundred messages within a given chat and who satisfied this activity threshold in at least two distinct chats. This constraint ensured that the users retained for analysis were active participants rather than occasional contributors, allowing us to compute stable estimates of their behavior and to evaluate how it varied across different contexts.

After applying these filters, each dataset was transformed into an aggregated structure that captured the interactions between individual users and their corresponding chats, expressed through (chat toxicity, user toxicity) pairs. We summarize the resulting filtered datasets in Table 3. Overall, this preprocessing pipeline allowed us to focus on users with a sufficiently rich interaction history, ensuring accurate measurement of toxicity patterns and enabling meaningful cross-community comparisons. Additional details on the resulting filtered datasets are reported in Table S2 and Figure S2 of the Supplementary Information.

**Table 3:** Aggregated dataset after preprocessing. Only users with at least 100 messages in two or more chats are included (i.e., active users). Each toxicity pair corresponds to a (chat–toxicity, user–toxicity) combination.

Dataset	Active Users	Chats	Toxicity Pairs	Avg. Chat Toxicity ($\mu \pm \sigma$) (%)	Avg. User Toxicity ($\mu \pm \sigma$) (%)
English1	1, 740	465	4, 607	$2.47 \pm 2.72$	$4.15 \pm 4.54$
Russian	3, 202	544	7, 630	$1.37 \pm 1.52$	$2.00 \pm 4.54$
Italian1	461	88	1, 041	$1.69 \pm 1.47$	$1.45 \pm 1.85$
Portuguese	646	86	1, 578	$1.26 \pm 1.31$	$1.62 \pm 2.32$
Italian2	19, 332	3, 502	62, 424	$1.33 \pm 1.59$	$1.62 \pm 3.48$
English2	9, 327	1, 378	26, 726	$1.50 \pm 2.96$	$1.70 \pm 4.87$

### Formal definitions

To formalize the quantities used in the manuscript, let us start by defining two sets: the set of users $\mathcal {U}$, and the set of chats $\mathcal {C}$. Given a chat $c \in \mathcal {C}$, let $\mathbb {M}_c$ denote its set of messages and $\hat{\mathbb {M}}_c \subseteq \mathbb {M}_c$ the subset of toxic messages. The toxicity of chat *c* is defined as $\mathbb {T}(c):= \frac{\;|\hat{\mathbb {M}}_c|\;}{|\mathbb {M}_c|}$. Given a user $u \in \mathcal {U}$ and a chat $c\in \mathcal {C}$, let $\mathbb {M}_{u,c} \subseteq \mathbb {M}_c$ denote the messages sent by *u* in chat *c*, and $\hat{\mathbb {M}}_{u,c} \subseteq \mathbb {M}_{u,c}$ the toxic ones. The user toxicity in chat *c* is defined as $\mathbb {T}(u \mid c):= \frac{\;|\hat{\mathbb {M}}_{u,c}|\;}{|\mathbb {M}_{u,c}|}$.

Moreover, given a threshold *n*, we define the user *u* active in the chat *c* if and only if $\mid \mathbb {M}_{u,c} \mid \ge n$. In order to have meaningful analysis, we firstly restricted our dataset only to users active in chats. We then restricted the dataset to users active in at least two different chats. Thus, given a threshold $n \in \mathbb {N}$, we define $\mathcal {U}_n$ as the set of users included in the analysis, and $\mathcal {D}_n$ as the dataset used for the analysis as

$$\begin{aligned} \mathcal {U}_n := \left\{ u \in \mathcal {U} \;:\; \left| \left\{ c \in \mathcal {C} \;:\; |\mathbb {M}_{u,c}| \ge n \right\} \right| \ge 2 \right\} \;\;\;\;\; \mathcal {D}_n := \left\{ (u,c) \in \mathcal {U}_n \times \mathcal {C} \;:\; |\mathbb {M}_{u,c}| \ge n \right\} . \end{aligned}$$

Then, for each user $u \in \mathcal {U}_n$, we denote by $\mathcal {C}_n(u):= \{ c \in \mathcal {C}: (u,c) \in \mathcal {D}_n \}$ the set of chats in which *u* is active. For each unordered pair $\{c_i,c_j\} \subseteq \mathcal {C}_n(u)$ with $c_i \ne c_j$, let $c^{+},c^{-} \in \{c_i,c_j\}$ be such that $\mathbb {T}(c^{+}) \ge \mathbb {T}(c^{-})$. In the case $\mathbb {T}(c_i)=\mathbb {T}(c_j)$, ties are broken using the chat identifier. We then define the variation in user toxicity as a function of the variation in chat toxicity

$$\begin{aligned} \Delta _u(\{c_i,c_j\}) := \left( \mathbb {T}(c^{+}) - \mathbb {T}(c^{-}), \mathbb {T}(u \mid c^{+}) - \mathbb {T}(u \mid c^{-})\right) . \end{aligned}$$

Finally, we define the dataset used for Figure 3

$$\begin{aligned} \mathcal {S}_n := \left\{ \left( u,\{c_i,c_j\},\Delta _u(\{c_i,c_j\})\right) : u \in \mathcal {U}_n,\, \{c_i,c_j\} \subseteq \mathcal {C}_n(u),\, c_i \ne c_j \right\} . \end{aligned}$$

These formal definitions jointly define the core quantities used throughout the manuscript and underpin the subsequent analyses.

### Correlation measures between user and chat toxicity

As the datasets’ distributions span several orders of magnitude and display different tail behaviors, heavy-tailed or light-tailed, as shown in Figure 1, we employed logarithmic binning for visualization and analysis. Logarithmic binning reduces noise in sparsely populated regions of the distribution, while also stabilizing the estimates in the tails. Although it is classically applied to heavy-tailed data^44,72^, it is also appropriate for light-tailed or rapidly decaying distributions, as it provides a balanced representation^73^.

Each dataset was divided into 40 logarithmically spaced bins, and bins with fewer than 10 observations were discarded to avoid undersampling. We then computed the Pearson correlations using the midpoint of the bin borders and the mean value of the elements in each bin. We used the pearsonr method of the scipy package to compute both the correlation values, as well as the p-values. Moreover, to ensure that our results did not depend on the specific binning procedure, we repeated all analyses, varying the number of bins from 20 to 100 in steps of 10, and the minimum bin size threshold from 10 to 20 in steps of 2. A recap of this validation, for each analysis, can be found in Table 4. This table shows that the median values obtained from all validation runs for each analysis remain consistently high and comparable to the original results. In addition, the standard deviation is relatively low, indicating stable outcomes. The complete set of correlation values for all tested configurations is provided in Tables S4, S5, and S6 of the Supplementary Information.

**Table 4:** Robustness to binning choices. Median Pearson correlations ($\mu$) with standard deviation ($\sigma$) across validation runs varying the number of logarithmic bins (20–100) and the minimum bin size threshold (10–20, step 2). Columns correspond to the same analyses shown in Figures 2a, Figures 2b, and 3, confirming that correlations remain stable across binning configurations.

Dataset	Static (Figure 2a)	Leave-One-Out (Figure 2b)	Dynamic (Figure 3)
English1	$0.995 \pm 0.001$	$0.991 \pm 0.005$	$0.853 \pm 0.069$
Russian	$0.989 \pm 0.002$	$0.970 \pm 0.016$	$0.766 \pm 0.073$
Italian1	$0.978 \pm 0.012$	$0.946 \pm 0.034$	$0.801 \pm 0.106$
Portuguese	$0.982 \pm 0.007$	$0.949 \pm 0.047$	$0.885 \pm 0.069$
Italian2	$0.990 \pm 0.009$	$0.987 \pm 0.008$	$0.898 \pm 0.033$
English2	$0.996 \pm 0.002$	$0.989 \pm 0.008$	$0.989 \pm 0.005$

### Fitting user conformity patterns

To approximate the behavior of individual users, we fit a linear model relating the toxicity of each user in a chat to the overall toxicity of that chat. This was done across all chats in which the user participated, using the LinearRegression module from the scikit-learn package^74^. The resulting slope and intercept of the fitted line provide a characterization of the behavior of a user, as shown in Figure 4a. We conceptualize this slope as a conformity index, where $\theta$ denotes the regression slope expressed in angular degrees. We then define the slope $\theta$ as the inverse of the tangent of the angular coefficient, and thus we classify users according to the direction and magnitude of their slope:

- *Conformist users*: $\theta> \tau$ (i.e., toxicity increases as the environment becomes more toxic).
- *Anti-conformist users*: $\theta < -\tau$ (i.e., toxicity decreases as the environment becomes more toxic).
- *Independent users*: $|\theta | \le \tau$ (i.e., toxicity remains stable across contexts).
- *Zen users*: users who never displayed any toxicity in any chat.

A key methodological challenge is defining what counts as “near to zero” when distinguishing independent users from conformists or anti-conformists. Since raw regression coefficients lack an intuitive scale, we convert slope values into angular degrees, where $0^\circ$ corresponds to a flat line. This allows us to interpret thresholds as angular deviations from behavioral stability. In the main analysis, we set $\tau =15$: users whose slope lies between $-15^\circ$ and $+15^\circ$ are considered independent, while slopes exceeding that range indicate conformity or anti-conformity. This cutoff offers a reasonable trade-off between sensitivity and interpretability.

We then estimate the statistical variability of user type percentages using a nonparametric bootstrap procedure. We repeat this procedure separately for each dataset and for each minimum-activity threshold considered in the filtering procedure. Let *n* be the number of users in a given dataset, we generate $B = 1000$ bootstrap samples composed of *n* users extracted from the dataset with replacement. This way, the sample size matches the original dataset size. For each bootstrap sample we compute the percentage of each user type. We then summarize the resulting distribution across samples by its mean and standard deviation. Figure 4c includes the uncertainty bands corresponding to the bootstrap mean ± one standard deviation.

**Fig. 5:**
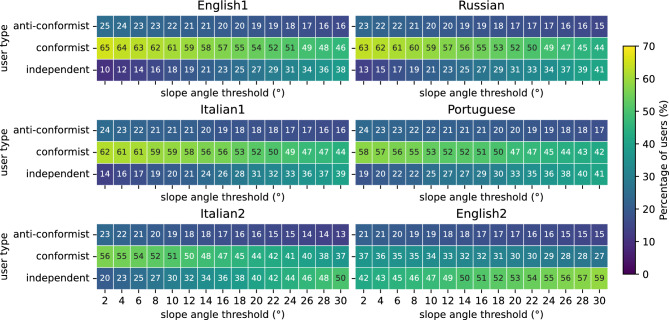
Sensitivity analysis of user classification as a function of the slope angle threshold $\tau$. The plot shows how the proportions of conformist, independent, and anti-conformist users vary as the threshold used to define independence is adjusted from $2^\circ$ to $30^\circ$. Users are classified as independent when $|\theta | < \tau$, where $\theta$ is the regression slope expressed in angular degrees. Despite changes in the threshold, the ratio between conformists and anti-conformists remains approximately stable, indicating that the predominance of conformist behavior is robust across a broad range of cutoff values.

Finally, to assess the robustness of our findings, we systematically vary the cutoff used to determine whether a user’s slope is sufficiently close to zero to be considered independent. Specifically, we test a wide range of angular thresholds, treating users as independent whenever their absolute slope angle satisfies $|\theta | < \tau$, with $\tau$ ranging from $2^\circ$ to $30^\circ$ (Figure 5). Across the entire threshold range, the relative proportions of conformist, independent, and anti-conformist users remain qualitatively stable, indicating that the prevalence of conformist behavior is not an artifact of a particular cutoff choice and that the relative balance between conformist and anti-conformists remains stable across datasets.

## Supplementary Information

Supplementary Information.

## Data Availability

In this study, we leveraged three datasets: The Pushshift Telegram Dataset, a multilingual resource published by Baumgartner et al. and available at https://doi.org/10.5281/zenodo.3607497; an Italian dataset introduced by Alvisi et al. in their study (10.48550/arXiv.2504.19594), which will be made publicly available upon publication of the associated paper;  and a novel English dataset that we collected and released as part of this work, available at https://data.d4science.net/1rLI4.
