# Supplementary material for: From toxicity to conformity: adaptive user behavior to social norms in Telegram communities
Source: Sci Rep. 2026 Apr 24;16:18907. doi: 10.1038/s41598-026-49756-w (PMC13276170; doi:10.1038/s41598-026-49756-w)
Supplement: Supplementary file 1 — Supplementary Information. [file 41598_2026_49756_MOESM1_ESM.pdf]

scientific reports

Supplementary Information for

From toxicity to conformity: adaptive user behavior to social norms in Telegram communities

L. Alvisi, V. Popa, G. Cola, S. Tardelli, M. Tesconi

Corresponding author: V. Popa.
E-mail: victoria.popa@phd.unipi.it

This PDF file includes:

Supplementary Information
Figs. S1 to S2
Tables S1 to S9
SI References

Supplementary Information

Results

Preliminaries

User selection

User participation. We report the empirical distribution of the number of chats per user in Figure S1a, showing the frequency distribution. As mentioned, we consider users who participated in at least two chats with at least 100 messages in each chat. As shown, user participation follows a steep heavy tailed distribution, with the vast majority of users participating in only a small number of chats (median = 2 across datasets, as we also report in Table S1). To more clearly characterize the tail of the distribution and assess the potential impact of highly multi-chat users (i.e., users participating in many chats), we additionally show the distribution of the number of chats per user as a complementary cumulative distribution function (CCDF), reported here in Figure S1b. The CCDF highlights that, across all datasets, user participation is strongly tailed but concentrated at low values. Users participating in more than 10 chats represent a small minority of the population (i.e., 0–2.4%, depending on the dataset, as reported in Table S3). Importantly, we further quantify their contribution to the total number of chat pairs. In fact, as the reviewer noted, since the number of chat pairs generated by a user scales as $\binom{k}{2}$, where k is the number of chats in which the user participates, the distribution of user participation directly determines whether pairwise analyses are broadly distributed or dominated by highly multi-chat users. Taking this point into account, we find that users with more than 10 chats account for only 0.4%–9.4% of all chat pairs, depending on the dataset (i.e., `English1`: 4.1%, `Russian`: 1.2%, `Italian1`: 0.4%, `Portuguese`: 1.2%, `Italian2`: 9.4%, `English2`: 5.8%, as reported in Table S3). Thus, although the number of pairs per user increases quadratically with participation, highly multi-chat users do not dominate the pairwise analysis.

Individual patterns of user norm conformity

Robustness of the conformity index. Given the number of observations, conventional regression-based significance tests would be challenging. As such, to address this concern, rather than relying on p-values, we provide an assessment of the robustness of the conformity index. Specifically, we conducted an additional robustness analysis using a leave-one-chat-out sensitivity procedure. For each user participating in at least three chats, we refitted the conformity regression after removing one chat at a time. Each refit produced a behavioral category assignment based on the slope, and we then assessed whether the majority of these assignments matched the user's original category. We report the results in Table S8.

In particular, results on this new analysis show that:

- Conformist users are consistently classified across datasets and configurations, remaining correctly classified in 98.7–100% of refits, indicating that conformist behavior is highly robust and not driven by individual influential chats.
- Anti-conformist users are also consistently classified, although with slightly lower stability (i.e., 78.95–92.4%).
- Independent users exhibit greater variability and are more frequently reclassified, often shifting toward the conformist category. This pattern suggests that many users classified as independent lie close to the boundary between independence and weak positive conformity, thus interpret results concerning independent users with caution and as exploratory.
- Zen users remain in the same category by definition.

Consistently, when restricting the analysis to users participating in at least four chats, classification stability increases substantially, further suggesting robustness of the findings.

Zen users. The higher proportion of zen users observed in the `English2` dataset may be explained by several factors related to the data collection strategy and the thematic composition of the dataset. In particular, the `English2` dataset was collected starting from a seed of Telegram chats obtained from *tgstat.com*, specifically including English-language channels catalogued on the platform, along with their associated discussion groups retrieved from them. Since *tgstat.com* also provides a topic label for each listed chat, it was possible to inspect the distribution of chats by topic. This analysis shows that the `English2` dataset is more thematically homogeneous and largely centered on cryptocurrency-related discussions, which account for approximately 51% of the seed chats. By comparison, the second largest category (Politics) represents only 7%, with the remaining topics each accounting for only a small fraction of the dataset. By contrast, other datasets used in the study include a broader variety of topics and communities. For example, the `Italian2` dataset covers multiple thematic domains across Telegram communities by design (1). More generally, the greater heterogeneity of the other datasets is largely due to the data collection strategy: they were built using a snowball sampling approach that progressively included additional chats discovered through message forwarding links, thereby increasing the diversity of topics beyond the initial seeds. In contrast, the snowball strategy was not applied to the `English2` dataset. For this dataset, we retained only the initial seed chats for practical reasons related to the large volume of data already collected and the availability of several other datasets in the study. As a consequence, `English2` remained more topically concentrated than the other datasets.

This difference in topical diversity may influence the observed distribution of behavioral categories. In particular, communities centered on cryptocurrency discussions often involve informational or promotional messages (e.g., token announcements, trading

signals, or project updates) (2–4) rather than contentious debates. As a consequence, discussions in these contexts may naturally display lower levels of toxic language compared to more politically or socially contentious environments, which may in turn result in a higher proportion of users classified as zen (i.e., users who consistently produced no toxic messages across all chats in which they participated). Consistent with this interpretation, previous analyses of `Italian2` dataset indicate that cryptocurrency-related communities tend to exhibit relatively low levels of toxicity (1). Indeed, bots typically display repetitive or promotional messaging patterns (5). Accordingly, we implemented several steps in our preprocessing pipeline to minimize the presence and influence of bots. During data cleaning, we removed system notifications, service messages, and bot-generated messages using Telegram metadata fields indicating automated accounts (e.g., `from_user.is_bot`, `from_user.is_scam`, `from_user.is_fake`, etc.). Nevertheless, we acknowledge that no filtering strategy can completely eliminate automated or semi-automated accounts and addressing this is beyond the main scope of this study. In addition, because the `English2` dataset covers recent data (i.e., 2024), it is possible that the increasing adoption of LLM-based tools and more sophisticated automated agents may also affect the reliability of Telegram's automated account identification mechanisms. For these reasons, we cannot completely rule out the presence of spam or automated activity that might partially explain the higher proportion of zen users, especially given the interaction patterns typical of cryptocurrency communities.

Methods

Data preprocessing

Perspective API Labeling. We report the total number of messages processed and the number of messages successfully labeled by the Perspective API for each dataset. These statistics are now reported in Table S7.

Chat size. We provide additional information on the typical size of the groups, specifically of those groups considered in the analysis after applying the user-activity filtering criteria (i.e., chats retained only if they contain users who satisfy the activity criterion of ≥100 messages in at least two chats). In particular, we report the average size of the chats included in the analysis, as this information may help contextualize the results. Group size is computed considering all users participating in the selected chats, rather than only the subset of users retained for the behavioral analysis. Table S2 reports summary statistics of the number of unique users per chat. The median values indicate that most groups contain a few hundred users, while the interquartile range (IQR) shows that the central portion of the distribution spans several hundred users across all datasets. This suggests that most groups are of moderate size, whereas larger groups occur less frequently. The dataset `English2` contains larger groups than the other datasets, as indicated by the higher median and wider IQR. Figure S2 reports the complementary cumulative distribution function of the number of unique users per chat. The curves decrease smoothly over a wide range of values, indicating that small and medium-sized groups are common while very large groups are comparatively rare. The curves for `English1`, `Italian1`, `Portuguese`, `Russian`, and `Italian2` follow a similar pattern, whereas `English2` extends toward larger values, consistent with the larger group sizes reported in Table S2. Together, such Table and Figure describe the variability and range of group sizes in the datasets.

Correlation measures between user and chat toxicity

Robustness to binning choices. To ensure that our results did not depend on the specific binning procedure, we repeated all analyses, varying the number of bins from 20 to 100 in steps of 10, and the minimum bin size threshold from 10 to 20 in steps of 2. The complete set of correlation values for all tested configurations is provided in Tables S4, S4, and S6. The highlighted row corresponds to the configuration used in the main paper.

Fitting user conformity patterns

Statistics of behavioral groups. In Table S9, we report the number of users and the total number of messages produced by each behavioral category across datasets. These statistics provide an overview of the relative size and activity levels of the different behavioral groups. In particular, we report group size and activity as descriptive indicators.

Dataset	Median chats/user	Mean chats/user	Std. dev.
`English1`	2	2.65	1.69
`Russian`	2	2.38	1.27
`Italian1`	2	2.26	0.64
`Portuguese`	2	2.44	0.95
`Italian2`	2	3.23	2.75
`English2`	2	2.87	2.69

Table S1. Distribution of chats per user. For each dataset, we report the median, mean, and standard deviation of the number of chats each user participates in.

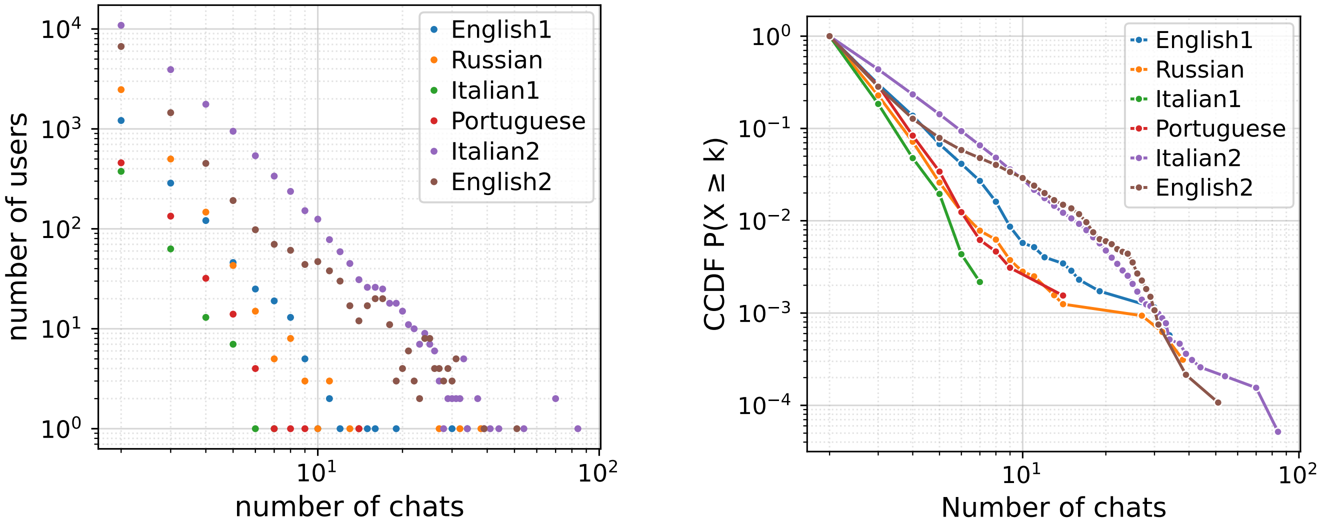

(a) Empirical distribution of the number of chats per user.

(b) Complementary cumulative distribution function (CCDF) of chats per user.

Fig. S1. Distribution of chats per user. Distribution of the number of chats per user. (a) shows discrete frequency distribution, while (b) shows the CCDF highlighting the heavy-tailed participation pattern.

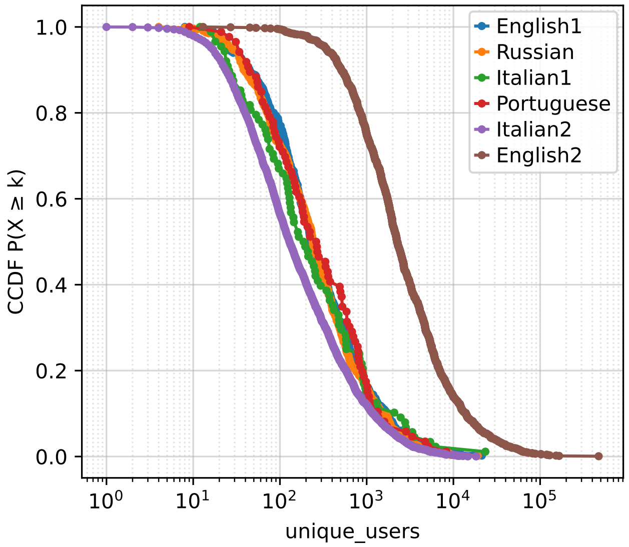

Fig. S2. CCDF of the number of unique users per chat across the analyzed datasets. Most chats contain a few hundred users, while very large groups occur less frequently. The dataset `English2` extends toward larger values, indicating the presence of substantially larger groups compared to the other datasets.

Dataset	Mean (chat size)	Std. dev.	Median (chat size)	IQR
`English1`	742.75	1,882.60	229.0	522.00
`Russian`	654.46	1,415.74	238.5	494.25
`Italian1`	860.38	2,660.84	176.0	513.25
`Portuguese`	659.66	1,214.50	242.0	686.75
`Italian2`	498.31	1,174.00	128.5	377.00
`English2`	6,565.54	18,235.06	2,240.5	4,659.00

Table S2. Summary statistics of chat sizes in terms of users (filtered chats). The interquartile range (IQR) is defined as the difference between the 75th and 25th percentiles.

Dataset	Users (%) (users in $\geq$ 10 chats)	User–chat pairs (%) (users in $\geq$ 10 chats)
`English1`	0.52	4.14
`Russian`	0.25	1.25
`Italian1`	0.00	0.43
`Portuguese`	0.16	1.24
`Italian2`	2.16	9.35
`English2`	2.40	5.83

Table S3. Contribution of high-participation users (≥ 10 chats) to total chat pairs. For each dataset, we report the percentage of users with at least 10 chats and the percentage of user–chat pairs contributed by these users.

English1	Russian	Italian1	Portuguese	Italian2	English2	Bins	Elements per bin
0.995063	0.987788	0.993885	0.988803	0.998501	0.999220	20	10
0.994910	0.987788	0.993885	0.988803	0.998501	0.999220	20	12
0.994910	0.987788	0.993885	0.988803	0.998501	0.999220	20	14
0.994910	0.987788	0.993885	0.988803	0.998501	0.999220	20	16
0.994910	0.987788	0.993128	0.988376	0.998501	0.999208	20	18
0.994910	0.987788	0.993128	0.988376	0.998501	0.999208	20	20
0.997393	0.992245	0.988992	0.983810	0.998514	0.997667	30	10
0.997331	0.992245	0.988992	0.983810	0.998514	0.997643	30	12
0.997331	0.992245	0.988992	0.983588	0.998503	0.997643	30	14
0.997331	0.992182	0.988992	0.983588	0.998491	0.997643	30	16
0.997331	0.992182	0.987153	0.993139	0.998491	0.997643	30	18
0.997331	0.992080	0.987153	0.993139	0.998491	0.997643	30	20
0.997551	0.992640	0.987776	0.983291	0.998818	0.999163	40	10
0.997499	0.992640	0.991311	0.983291	0.998818	0.999163	40	12
0.997499	0.992640	0.991311	0.983052	0.998820	0.999163	40	14
0.997499	0.992542	0.990646	0.983251	0.998822	0.999163	40	16
0.997499	0.992542	0.989639	0.992513	0.998512	0.999163	40	18
0.997499	0.992454	0.989639	0.992513	0.999020	0.999163	40	20
0.995606	0.988805	0.961261	0.978993	0.985084	0.993073	50	10
0.995606	0.988805	0.985046	0.978993	0.985084	0.992970	50	12
0.995547	0.988805	0.988643	0.978604	0.984971	0.992970	50	14
0.995556	0.988611	0.987984	0.978604	0.984849	0.992970	50	16
0.995484	0.988611	0.986621	0.987103	0.984849	0.992970	50	18
0.995407	0.988473	0.986621	0.986946	0.986800	0.992970	50	20
0.996843	0.990924	0.961146	0.983236	0.996224	0.994660	60	10
0.996775	0.990924	0.980667	0.983236	0.996224	0.994660	60	12
0.996775	0.990845	0.979991	0.983006	0.996215	0.994660	60	14
0.996779	0.990679	0.979024	0.982788	0.996088	0.994629	60	16
0.996779	0.990612	0.981296	0.989735	0.996070	0.994629	60	18
0.996740	0.990500	0.981296	0.989735	0.998864	0.994629	60	20
0.995092	0.988962	0.950710	0.981971	0.989687	0.995924	70	10
0.994997	0.988962	0.974918	0.981971	0.989626	0.995859	70	12
0.994961	0.988962	0.974070	0.981689	0.989493	0.995859	70	14
0.994940	0.988768	0.972810	0.981663	0.989493	0.995859	70	16
0.994860	0.988540	0.970666	0.988787	0.989424	0.995859	70	18
0.994797	0.988540	0.970666	0.988723	0.987146	0.995859	70	20
0.994898	0.990516	0.948604	0.980067	0.986698	0.993340	80	10
0.994800	0.990516	0.972001	0.980067	0.986551	0.993340	80	12
0.994784	0.990516	0.970694	0.979821	0.986388	0.993340	80	14
0.994776	0.990293	0.968946	0.979513	0.986202	0.993340	80	16
0.994776	0.990197	0.965658	0.987007	0.986202	0.993305	80	18
0.994776	0.990197	0.966431	0.987007	0.982338	0.993305	80	20
0.994399	0.989011	0.952343	0.976332	0.987778	0.995571	90	10
0.994396	0.988958	0.975581	0.976332	0.987778	0.995571	90	12
0.994291	0.988878	0.974829	0.975413	0.987649	0.995552	90	14
0.994176	0.988569	0.973668	0.975638	0.987649	0.995552	90	16
0.994176	0.988455	0.975338	0.982513	0.987581	0.995533	90	18
0.994067	0.988455	0.976283	0.982669	0.984656	0.995369	90	20
0.993135	0.984476	0.958415	0.966101	0.970102	0.994794	100	10
0.993056	0.984427	0.975878	0.965538	0.969966	0.994772	100	12
0.992873	0.984304	0.974999	0.964383	0.969675	0.994749	100	14
0.993613	0.983884	0.973746	0.968271	0.968061	0.994726	100	16
0.993506	0.983884	0.974437	0.974177	0.968061	0.994726	100	18
0.993506	0.983722	0.975236	0.974070	0.996867	0.994726	100	20

Table S4. Pearson correlation coefficients for the analysis shown in Figure 2a under different binning configurations. Each row corresponds to a different combination of the number of logarithmic bins and the minimum number of elements per bin used in the robustness analysis. The highlighted row indicates the configuration used in the main analysis shown in Figure 2a.

English1	Russian	Italian1	Portuguese	Italian2	English2	Bins	Elements per bin
0.994220	0.988918	0.987978	0.984611	0.995005	0.998351	20	10
0.994220	0.988673	0.987978	0.984611	0.995005	0.998351	20	12
0.994220	0.988673	0.987978	0.984611	0.995005	0.998351	20	14
0.994220	0.988673	0.988705	0.983970	0.995005	0.998351	20	16
0.994220	0.988673	0.988705	0.983970	0.995005	0.998341	20	18
0.994220	0.988673	0.988705	0.983970	0.995005	0.998341	20	20
0.997556	0.982877	0.973579	0.976669	0.992212	0.996256	30	10
0.997556	0.982877	0.973579	0.975860	0.992212	0.996211	30	12
0.997556	0.982877	0.973579	0.975860	0.992212	0.996211	30	14
0.997556	0.982724	0.972403	0.975860	0.992048	0.996211	30	16
0.997556	0.982724	0.971189	0.976146	0.992048	0.996211	30	18
0.997556	0.982724	0.971189	0.976124	0.992048	0.996211	30	20
0.996287	0.951780	0.977962	0.973572	0.994289	0.997507	40	10
0.996287	0.951780	0.981684	0.972817	0.994289	0.997507	40	12
0.996287	0.951780	0.981265	0.972791	0.994310	0.997507	40	14
0.996295	0.990695	0.979679	0.973077	0.994254	0.997507	40	16
0.996295	0.990695	0.975528	0.972640	0.994254	0.997507	40	18
0.996295	0.990695	0.974375	0.972625	0.994254	0.997507	40	20
0.982649	0.984471	0.925058	0.977385	0.991670	0.971596	50	10
0.982364	0.984471	0.931817	0.978103	0.991670	0.978542	50	12
0.982364	0.984471	0.928429	0.979688	0.991636	0.978542	50	14
0.981842	0.984344	0.913280	0.979688	0.991605	0.978542	50	16
0.981842	0.984344	0.915350	0.979989	0.991605	0.978542	50	18
0.981842	0.984229	0.915350	0.980577	0.991605	0.978542	50	20
0.992605	0.980671	0.930633	0.963043	0.985304	0.987048	60	10
0.992605	0.980671	0.936818	0.962898	0.985304	0.987048	60	12
0.992605	0.980671	0.933652	0.962048	0.985237	0.991798	60	14
0.992452	0.980433	0.928033	0.960858	0.985142	0.991798	60	16
0.992275	0.980433	0.960536	0.960707	0.985142	0.991798	60	18
0.992157	0.980293	0.958495	0.961027	0.985142	0.991798	60	20
0.994352	0.964048	0.902417	0.783310	0.987686	0.960656	70	10
0.994408	0.964048	0.909527	0.816941	0.987686	0.994609	70	12
0.994375	0.964048	0.982164	0.816941	0.987609	0.994609	70	14
0.994369	0.963720	0.978890	0.806990	0.987609	0.994609	70	16
0.994391	0.963040	0.974445	0.851136	0.987609	0.994609	70	18
0.994210	0.963040	0.974445	0.937014	0.987609	0.994609	70	20
0.989515	0.960850	0.907742	0.931265	0.988724	0.981925	80	10
0.989294	0.960850	0.905891	0.931265	0.988704	0.986618	80	12
0.989069	0.960585	0.951711	0.931234	0.988629	0.986618	80	14
0.989069	0.960242	0.952464	0.930474	0.988629	0.986618	80	16
0.988604	0.959553	0.952464	0.929170	0.988605	0.986547	80	18
0.988604	0.970693	0.952464	0.924446	0.988643	0.986474	80	20
0.990206	0.953992	0.896556	0.931662	0.969619	0.984940	90	10
0.990102	0.946685	0.893221	0.933231	0.969619	0.984805	90	12
0.990024	0.945904	0.942364	0.963717	0.969097	0.984805	90	14
0.990074	0.944629	0.941834	0.963519	0.969097	0.984805	90	16
0.989965	0.944073	0.941834	0.963519	0.969097	0.984734	90	18
0.990045	0.943534	0.941834	0.960940	0.969097	0.984660	90	20
0.983505	0.949650	0.858565	0.923728	0.977633	0.982978	100	10
0.983005	0.949003	0.871719	0.950849	0.977445	0.982770	100	12
0.984629	0.948591	0.913983	0.952313	0.977372	0.982696	100	14
0.984417	0.947333	0.912761	0.951561	0.981204	0.982696	100	16
0.984260	0.961238	0.900815	0.951708	0.981204	0.982696	100	18
0.984116	0.954538	0.900815	0.950459	0.982624	0.982696	100	20

Table S5. Pearson correlation coefficients for the analysis shown in Figure 2b under different binning configurations. Each row corresponds to a different combination of the number of logarithmic bins and the minimum number of elements per bin used in the robustness analysis. The highlighted row indicates the configuration used in the main analysis shown in Figure 2b.

English1	Russian	Italian1	Portuguese	Italian2	English2	Bins	Elements per bin
0.975693	0.776022	0.974570	0.988450	0.959964	0.998456	20	10
0.979243	0.782032	0.909152	0.988450	0.959964	0.998456	20	12
0.986746	0.782032	0.909152	0.988450	0.959964	0.998456	20	14
0.986746	0.782032	0.909152	0.988450	0.959964	0.998450	20	16
0.986746	0.782032	0.909152	0.988450	0.959964	0.998450	20	18
0.988083	0.782032	0.909152	0.987855	0.959964	0.998450	20	20
0.853793	0.680253	0.893501	0.956576	0.930552	0.988130	30	10
0.853793	0.676122	0.893501	0.956576	0.930445	0.988130	30	12
0.853793	0.676122	0.893501	0.956576	0.930445	0.991760	30	14
0.853793	0.949224	0.893501	0.934259	0.930445	0.991760	30	16
0.853793	0.949224	0.893501	0.934259	0.930445	0.991760	30	18
0.850863	0.949224	0.893501	0.930332	0.930445	0.991748	30	20
0.866155	0.831138	0.819395	0.950374	0.928441	0.992718	40	10
0.864513	0.832040	0.819395	0.950374	0.928090	0.992718	40	12
0.864513	0.829853	0.831322	0.950374	0.928090	0.992691	40	14
0.864513	0.829853	0.842378	0.927857	0.928090	0.992691	40	16
0.862294	0.834717	0.842378	0.925719	0.928090	0.992691	40	18
0.862294	0.834717	0.842378	0.921702	0.928090	0.992680	40	20
0.790505	0.738900	0.864768	0.947989	0.892257	0.984633	50	10
0.790505	0.738900	0.872669	0.951296	0.892257	0.984633	50	12
0.790505	0.738900	0.859354	0.929327	0.892257	0.984576	50	14
0.791993	0.738900	0.869902	0.928787	0.892257	0.984576	50	16
0.791993	0.737187	0.615922	0.926602	0.891710	0.984576	50	18
0.788830	0.737187	0.615922	0.906803	0.891710	0.984565	50	20
0.804797	0.714261	0.836827	0.883137	0.864664	0.989540	60	10
0.803338	0.714261	0.855429	0.883137	0.864664	0.989540	60	12
0.803894	0.854014	0.855429	0.880814	0.865017	0.989473	60	14
0.803894	0.854014	0.848528	0.890836	0.865017	0.989473	60	16
0.799745	0.852526	0.848528	0.888093	0.865017	0.989473	60	18
0.773682	0.855358	0.816223	0.882225	0.865017	0.989442	60	20
0.824307	0.710479	0.829049	0.829509	0.906410	0.978082	70	10
0.824307	0.701934	0.842567	0.824412	0.905995	0.988657	70	12
0.791137	0.701713	0.550864	0.822026	0.905995	0.988622	70	14
0.789894	0.698429	0.659245	0.822026	0.868409	0.988622	70	16
0.784844	0.695431	0.739320	0.816887	0.868409	0.988622	70	18
0.780472	0.695431	0.723756	0.866262	0.868409	0.988601	70	20
0.832865	0.766986	0.775788	0.850056	0.885989	0.987763	80	10
0.835151	0.766986	0.827810	0.847922	0.885989	0.987763	80	12
0.817141	0.766789	0.833371	0.850611	0.885536	0.987710	80	14
0.814819	0.760985	0.861316	0.847403	0.885233	0.987710	80	16
0.812942	0.759011	0.714462	0.832022	0.885233	0.987710	80	18
0.969763	0.863822	0.690947	0.755115	0.885396	0.987687	80	20
0.807256	0.633288	0.854517	0.827768	0.875324	0.990849	90	10
0.807256	0.634662	0.574087	0.839656	0.875324	0.987250	90	12
0.805315	0.712168	0.566520	0.836371	0.875621	0.987219	90	14
0.815214	0.714558	0.679676	0.847280	0.875621	0.987219	90	16
0.809586	0.713253	0.676382	0.727998	0.874751	0.987219	90	18
0.980465	0.713455	0.676382	0.734242	0.874970	0.987200	90	20
0.849862	0.696035	0.820346	0.818147	0.861738	0.982483	100	10
0.848993	0.700861	0.814943	0.824917	0.861738	0.982483	100	12
0.828334	0.776534	0.824175	0.872888	0.863044	0.982420	100	14
0.974185	0.774556	0.855640	0.831849	0.863254	0.982420	100	16
0.973822	0.773054	0.611131	0.780131	0.862563	0.982420	100	18
0.973560	0.773482	0.611131	0.776147	0.862563	0.982393	100	20

Table S6. Pearson correlation coefficients for the analysis shown in Figure 3 under different binning configurations. Each row corresponds to a different combination of the number of logarithmic bins and the minimum number of elements per bin used in the robustness analysis. The highlighted row indicates the configuration used in the main analysis shown in Figure 3.

Data source	Total messages	Labeled messages	Discarded	Labeled messages (%)
Pushift	317,224,715	220,312,912	96,911,803	69.45%
Italian2	186,809,126	164,608,761	22,200,365	88.12%
English2	238,923,774	172,630,658	66,293,116	72.25%

Table S7. Summary statistics of the preprocessing step, reporting the number of messages retained after Perspective API labeling for each dataset.

Dataset	Conformist (%)	Zen (%)	Anti-conformist (%)	Independent (%)
English1	99.08	100.00	92.42	56.64
Russian	99.04	100.00	84.95	59.16
Italian1	100.00	100.00	88.89	57.14
Portuguese	100.00	100.00	78.95	72.50
Italian2	99.24	100.00	85.24	70.90
English2	98.71	100.00	84.87	74.36

Table S8. Stability of user behavioral classification under the leave-one-chat-out sensitivity analysis. For each user participating in at least three chats, we refit the conformity regression after removing one chat at a time and reassigned the behavioral category based on the resulting slope. Values report the percentage of refits in which the reassigned class matches the user's original classification.

Dataset	Group	Users	Users (%)	Total messages	Avg messages/user
English1	Independent	251	14.43	795,859	3,171
English1	Anti-conformist	358	20.57	584,476	1,633
English1	Conformist	994	57.13	2,472,626	2,488
English1	Zen	137	7.87	118,438	865
	**Total**	**1,740**	**100.00**	**3,971,399**	**2,282**
Russian	Independent	483	15.08	1,896,390	3,926
Russian	Anti-conformist	607	18.96	1,737,037	2,862
Russian	Conformist	1,762	55.03	7,006,992	3,977
Russian	Zen	350	10.93	319,927	914
	**Total**	**3,202**	**100.00**	**10,960,346**	**3,423**
Italian1	Independent	61	13.23	315,968	5,180
Italian1	Anti-conformist	90	19.52	234,728	2,608
Italian1	Conformist	258	55.97	840,231	3,257
Italian1	Zen	52	11.28	50,131	964
	**Total**	**461**	**100.00**	**1,441,058**	**3,126**
Portuguese	Independent	76	11.76	310,232	4,082
Portuguese	Anti-conformist	135	20.90	346,704	2,568
Portuguese	Conformist	330	51.08	1,235,964	3,745
Portuguese	Zen	105	16.26	87,752	836
	**Total**	**646**	**100.00**	**1,980,652**	**3,066**
Italian2	Independent	3,409	17.63	16,888,925	4,954
Italian2	Anti-conformist	3,355	17.35	9,910,789	2,954
Italian2	Conformist	9,173	47.45	36,391,031	3,967
Italian2	Zen	3,395	17.57	4,455,746	1,312
	**Total**	**19,332**	**100.00**	**67,646,491**	**3,499**
English2	Independent	932	9.99	3,280,713	3,520
English2	Anti-conformist	1,652	17.71	3,586,385	2,171
English2	Conformist	2,980	31.95	5,384,465	1,807
English2	Zen	3,763	40.35	4,062,587	1,079
	**Total**	**9,327**	**100.00**	**16,314,150**	**1,749**

Table S9. Descriptive statistics of behavioral groups across datasets. Total messages correspond to messages produced by users included in the analysis (i.e., users with at least 100 messages in at least two chats).

References

1. L Alvisi, S Tardelli, M Tesconi, Mapping the Italian Telegram Ecosystem: Communities, Toxicity, and Hate Speech. *arXiv preprint arXiv:2504.19594* (2025).
2. I Kang, et al., Deciphering crypto twitter in *Proceedings of the 16th ACM Web Science Conference.* pp. 331–342 (2024).
3. E Liu, et al., Give and take: An end-to-end investigation of giveaway scam conversion rates in *Proceedings of the 2024 ACM on Internet Measurement Conference.* pp. 704–712 (2024).
4. J Xu, B Livshits, The anatomy of a cryptocurrency {Pump-and-Dump} scheme in *28th USENIX Security Symposium (USENIX Security 19).* pp. 1609–1625 (2019).
5. K Lee, B Eoff, J Caverlee, Seven months with the devils: A long-term study of content polluters on twitter in *Proceedings of the international AAAI conference on web and social media.* Vol. 5, pp. 185–192 (2011).
